# pH dependent electro-oxidation of arsenite on gold surface: Relative kinetics and sensitivity

**DOI:** 10.1016/j.heliyon.2023.e14192

**Published:** 2023-03-06

**Authors:** Mohebul Ahsan, Muhammad Zobayer Bin Mukhlish, Nazia Khatun, Mohammad A. Hasnat

**Affiliations:** a Electrochemistry and Catalysis Research Laboratory (ECRL), Department of Chemistry, School of Physical Sciences, Shahjalal University of Science and Technology, Sylhet 3114, Bangladesh; b Department of Chemical Engineering and Polymer Science, Shahjalal University of Science & Technology, Sylhet 3114, Bangladesh; c Industrial Physics Division, Bangladesh Council of Scientific and Industrial Research (BCSIR), Bangladesh

**Keywords:** pH study, Arsenic oxidation, Au electrode, Kinetics, Sensitivity

## Abstract

A detailed kinetic investigation of As(III) oxidation was performed on gold surface within pH between ∼3.0 and ∼9.0. It was found that the As(III) oxidation on the gold surface follows a purely adsorption-controlled process irrespective of pH. The evaluated adsorption equilibrium constant decreased from 3.21 × 10^5^ to 1.61 × 10^5^ mol L^−1^ for acidic to basic medium, which implies the strong affinity of the arsenic species in the acidic medium. Besides, the estimation of Gibbs free energy revealed that an acidic medium promotes arsenic oxidation on gold surface. In mechanistic aspect, the oxidation reaction adopts a stepwise pathway for acidic medium and a concerted pathway for neutral and basic medium. From the substantial kinetic evaluation, it is established that a conducive and compatible environment for the oxidation of arsenic was found in an acidic medium rather than a basic or neutral medium on gold surface. Besides, in sensitivity concern, neutral and highly acidic medium is quite favourable for the arsenite oxidation on gold surface.

## Highlights

• A systematic kinetic investigation for arsenite oxidation was performed on gold surface from pH ∼3.0 to ∼9.0.
• The oxidation reaction proceeds through a purely adsorption-controlled process.
• The oxidation reaction follows stepwise mechanism for acidic medium and concerted mechanism for basic and neutral medium.
• The kinetic properties and sensitivity were determined for the arsenite oxidation reaction at different pH.

## Graphical abstract

**Figure:**
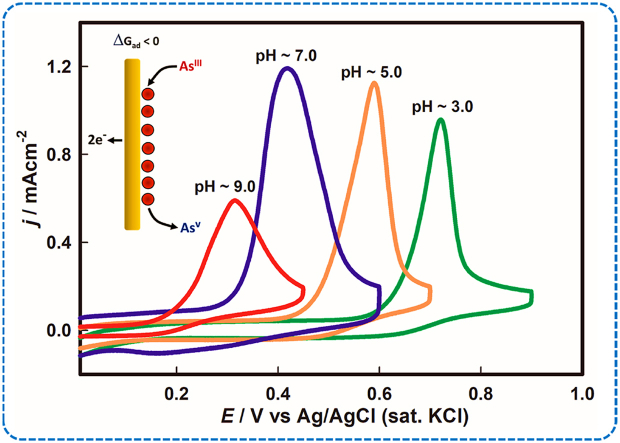

## 1 Introduction

Arsenic, a metalloid chemical present in groundwater, shares many qualities of metals. It is generally found on the surface and naturally occurs in air, water, soil, and foodstuffs. Anthropogenic contamination can boost its level to a certain extent [1,2]. Because of its function in complex biological and chemical activities, it is found in the environment in different forms of organic and inorganic chemicals. The two oxidation states, As(III) and As(V) are some of the most toxicologically relevant arsenic species out of four oxidation states [3]. Inorganic forms (arsenic trichloride, arsine and arsenic trioxide) of arsenic possess the deadly level of toxicity compared to organo-arsenic compounds by being sixty times more toxic than As(V) [4,5].

In general, many therapeutic or healing agents include arsenic as an essential constituent. Likewise, for the treatment of acute leukaemia, arsenic trioxide is being used. Due to its therapeutic usage, it can deactivate almost two hundred enzymes in our body that are actively engaged in cellular energy as well as DNA repairment pathways [6]. Recently, scientific communities have become concerned about the poisoning of the food chain by arsenic-laced water. As well as being primary source of drinking water, ground water contamination with arsenic also raises the eyebrows of people across the world [7,8]. Moreover, there remains a substantial amount of information pertaining to the cancer-causing role of arsenic and its link to a huge number of people suffering from critical diseases, as well as unfavourable skin consequences such as hyperkeratosis and depigmentation [5,9]. As a result of these findings, the US EPA has revised the acceptable limit of arsenic for safe drinking water to be 10 μgL^−1^ [10]. The situation regarding arsenic contamination in Bangladesh is a genuine concern because of the fact that more than thirteen thousand households are in jeopardy of surpassing the optimum level of arsenic standard for drinking water. The nationwide sporadic screening revealed that more than 80% of the villages tube wells were contaminated, which are termed “hotspots” in the southern and middle portions of the country. Consequently, the predominance of inorganic arsenic poses a significant risk factor to the health of the people of Bangladesh as well as global aspect [[11], [12], [13], [14], [15], [16], [17], [18], [19]].

Concerning this, sensors such as spectroscopic [[20], [21], [22], [23], [24], [25], [26]], nanobionic [26], optical [[27], [28], [29], [30]], and aptameric [[31], [32], [33], [34], [35]] are quite customary for all along arsenic detection. Aside from that, electrochemical detection is quite prominent due to its inherent advantages of portability, automation, highly sensitive detection, ease of preparation, and operational simplicity. Moreover, with the advancement of the nano-field, the synthesis of multivariant electrode materials is making it more feasible for selective and low limit of detection of arsenic. Loads of research has already been performed regarding the oxidation of arsenite on different types of electrodes [[36], [37], [38], [39], [40], [41]]. However, the kinetics of arsenite oxidation on Au surface at variable pH are yet to be studied. In this article, a detailed kinetic investigation along with sensitivity has been reported based on data received using voltammetric diagnosis at different pH.

## 2 Experimental

Sodium arsenite (NaAsO_2_), Disodium phosphate (Na_2_HPO_4_), Monosodium phosphate (NaH_2_PO_4_), Sulphuric acid (H_2_SO_4_), Sodium hydroxide (NaOH), Acetone (CH_3_)_2_CO, and Alumina powder (Al_2_O_3_) were brought from Merck, Germany. No additional purification was needed for the chemicals as of being analytical standard. Millipore Milli-Q water of 18.2 M Ω cm (Smart-Q30UT deionized water system, Qingdao, Shandong, China) was used to prepare all the required solutions. pH adjustment was achieved with suitable additions of acidic and basic buffer solutions.

All the experimental works were conducted with a CHI 660E electrochemical workstation (CH Instruments, USA) and Wave Driver 10 (Pine Research Instrumentation, USA) using typical three-electrode system. A gold (Au) disc electrode (0.2 mm of diameter) was employed for performing overall experimental actions. A counter electrode of Pt wire and Ag/AgCl (sat. KCl) were used as a reference electrode, respectively. All potentials here are referenced to the Ag/AgCl (sat. KCl) electrode. The surface of the gold electrode was polished and electrochemically cleaned, similar to the techniques described elsewhere in literature [42]. The total volume of the solution taken for each voltammetric experiment was 10 mL. Prior to the measurements, each solution was purged with N_2_ to stay out of any additional interferance.

## 3 Results and discussion

**Fig. 1:**
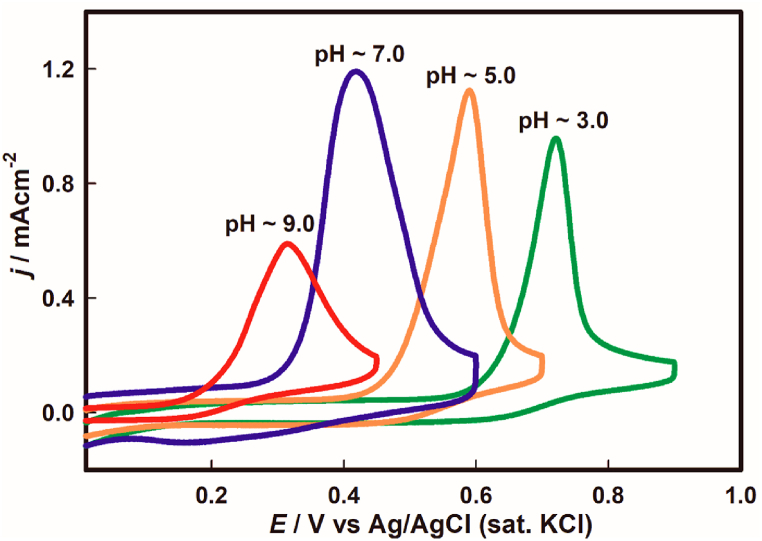
Cyclic voltammograms of 0.5 mM of As(III) oxidation on Au electrode surface at different pH values at 100 mVs^−1^ scan rate.

Cyclic voltammograms of the oxidation of 0.5 mM As(III) solution were recorded using a gold electrode under variable pH conditions from ∼3.0 to ∼9.0 at 100 mVs^−1^ scan rate. From Fig. 1, it is apparent that arsenic oxidation follows a pH dependent trend regarding potential switch during positive going scan and almost no subsequent reduction peak was observed in the reverse scanning.

Here, the potential has an interesting relationship pertaining to the oxidation of arsenic at the Au electrode from acidic to basic pH. Clearly, the electrostatic energy contribution is comparatively much higher for acidic pH than that of basic pH conditions in regards of As(III) oxidation. Moreover, a distinct comparison of the voltammetric features at different pH are tabulated in Table 1 for the 0.5 mM As(III) oxidation.

**Table 1:** Voltammetric properties of 0.5 mM of As(III) oxidation on Au electrode at different pH.

Voltammetric properties at different pH				
pH	*j* (mAcm^−2^)	*E*_*onset*_ (V)	*E*_*p*_ (V)	RSD^a^ (%)
3.0	0.96	0.58	0.72	2.63
5.0	1.12	0.45	0.59	2.86
7.0	1.19	0.27	0.41	3.01
9.0	0.60	0.15	0.31	2.12

a The value reflects the precision of three consecutive findings.

### 3.1 Effect of concentration

**Fig. 2:**
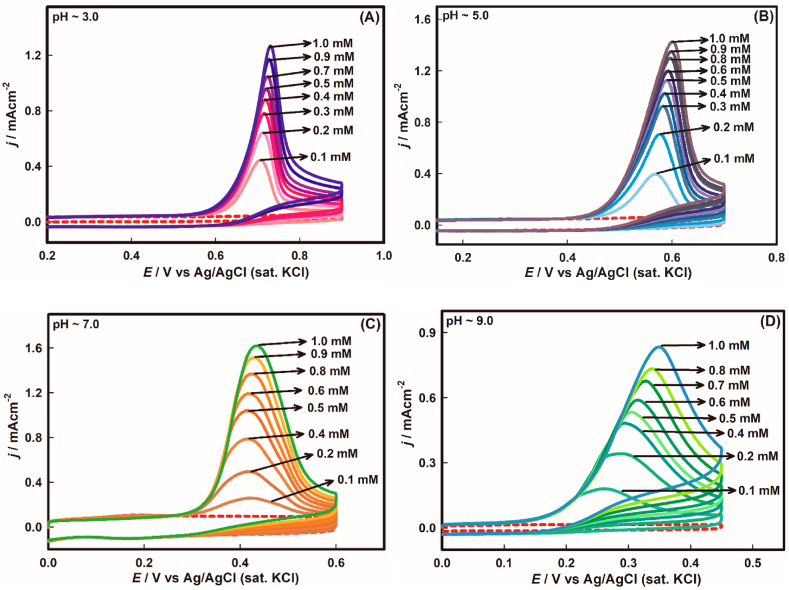
Concentration dependent CVs of As(III) oxidation on Au electrode surface at the pH value of (A) ∼3.0, (B) ∼5.0, (C) ∼7.0, and (D) ∼9.0 at 100 mVs^−1^ scan rate.

Concentration variant cyclic voltammograms of As(III) oxidation were recorded at different pH using Au electrode at 100 mVs^−1^ scan rate as can be seen from Fig. 2(A-D). Apparently in each case of pH, peak current density, *j*_*p*_, shoots up with the increment of As(III) concentration. It is noticeable that the peak potential has slight variation from pH ∼3.0 to ∼7.0 but in case of pH ∼9.0, a distinctive shift of potential is visible with the addition of As(III) concentration where concentration overpotential was needed. Moreover, the onset potential shifted to lower value from acidic to basic conditions pertaining to the As(III) oxidation. In general, the effects of concentration against potential have significant importance in kinetic appreciation. In this regard, the kinetic properties were unveiled using this effect in the later sections.

Later, the linear sweep voltammograms (see Fig. S1(A-D) of supplementary material) were recorded to check out the sensitivity at variable pH environment from the slope of the linear relationship of current density against concentration (see Fig. S2(A-D) of supplementary material). The sensitivity was found to be 1.4, 0.53, 4.78 and 0.76 mA cm^−2^ mM^−1^ for pH ∼3.0, ∼5.0, ∼7.0 and ∼9.0, respectively. That means for the oxidation of arsenite, gold electrode is highly sensitive at neutral medium and then at highly acidic medium. Besides, the electrode is not quite sensitive at pH ∼5.0 and ∼9.0, compared to other medium.

### 3.2 Effect of scan rates

**Fig. 3:**
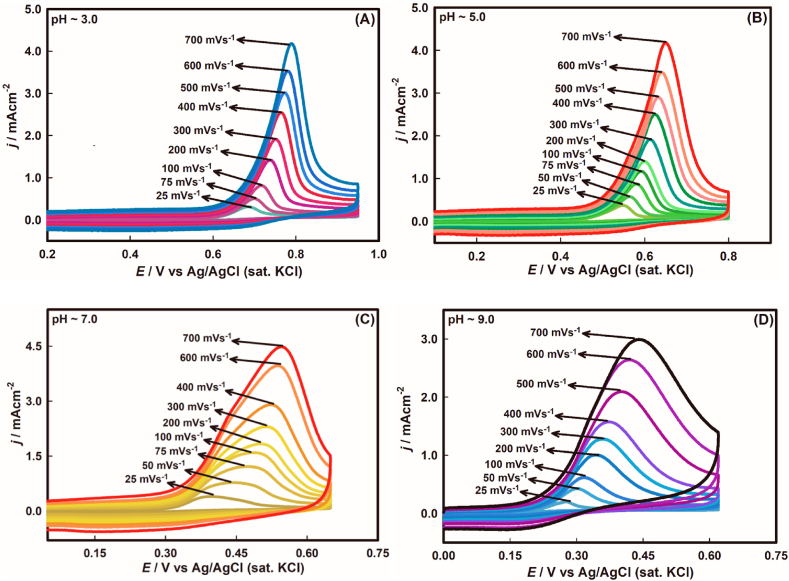
Scan rate dependent CVs of 1.0 mM of As(III) oxidation on Au electrode surface at variable pH.

To elucidate the intrinsic features of electrochemical reactions, especially kinetic and mechanistic aspect, effects of scan rate have crucial importance. Hence, the dependency of peak current density with respect to scan rate was recorded as shown in Fig. 3(A-D). It is discernible in the case of each pH value that peak current density amplifies with the consecutive increment of scan rates. This observation endorses the fact that the decrement of the diffusion layer thickness takes place with the increment of scan rates, which turns out to be the increment of current density.

**Fig. 4:**
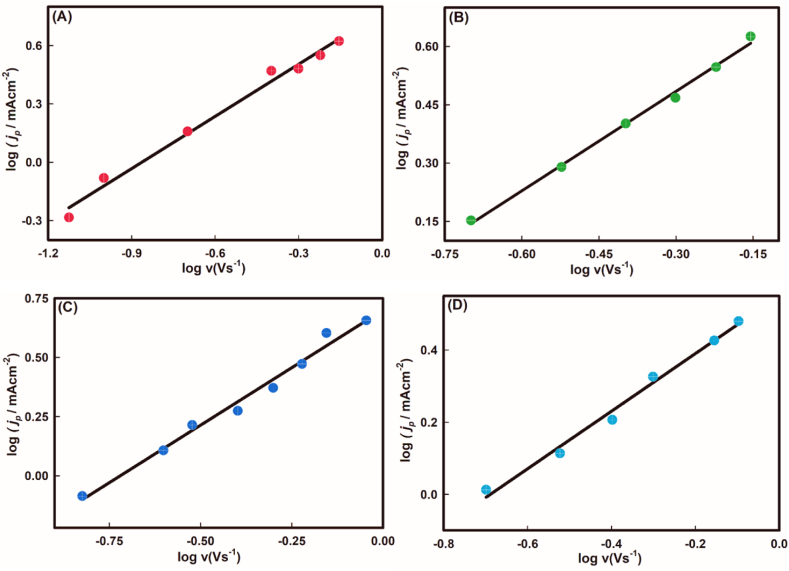
Plots of logarithm of current density, *j*_*p,*_ against logarithm of scan rate at variable pH values where (A) pH ∼3.0, (B) pH ∼5.0, (C) pH ∼7.0 and (D) pH ∼9.0.

Primarily, the impact of scan rate on kinetic behaviour was examined to assume the surface phenomena, whether diffusion or adsorption-controlled process. From the logarithm of current density against logarithm of scan rate (see Fig. 4(A-D)), the slope for consecutive pH values were found to be 0.89, 0.85, 0.97, 0.81 as given in eqs. (1), (2), (3), (4). Herein, the fractional values signify that the oxidation of As(III) on gold surface is typically an adsorption-controlled process where the electroactive species of As adsorbs from the bulk solution to the planar electrode surface [43].

Linear data fit yields the equations for the consecutive pH values,

(1) $$\log\;j_{p}\left({mAcm}^{-2}\right)=0.890\;\log v\left({Vs}^{-1}\right)+0.771;\;\left(r^{2}=0.99\right)$$

(2) $$\log\;j_{p}\left({mAcm}^{-2}\right)=0.850\;\log v\left({Vs}^{-1}\right)+0.740;\;\left(r^{2}=0.99\right)$$

(3) $$\log\;j_{p}\left({mAcm}^{-2}\right)=0.971\;\log v\left({Vs}^{-1}\right)+0.685;\;\left(r^{2}=0.99\right)$$

(4) $$\log\;j_{p}\left({mAcm}^{-2}\right)=0.810\;\log v\left({Vs}^{-1}\right)+0.556;\;\left(r^{2}=0.99\right)$$

### 3.3 Kinetics

**Fig. 5:**
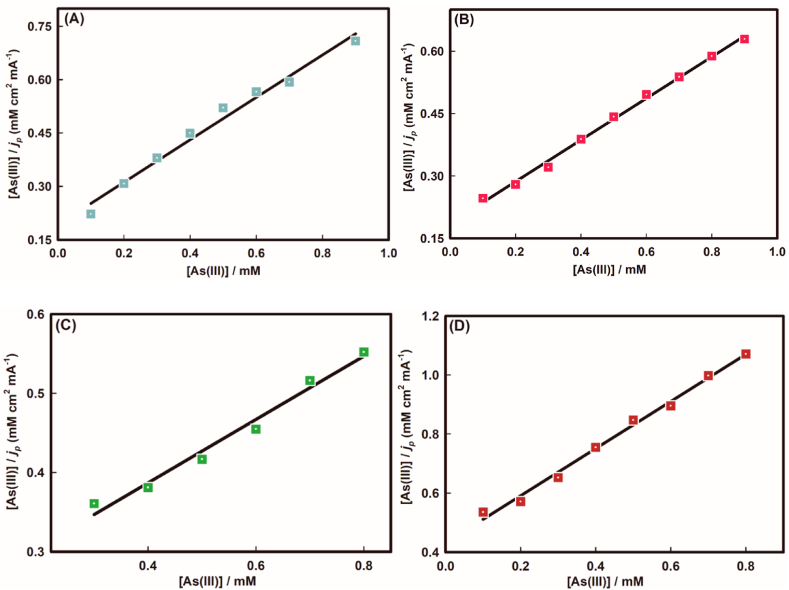
Plots of [As(III)]/*j*_*p*_, against [As(III)] using the modified Langmuir equation at (A) pH ∼3.0, (B) pH ∼5.0, (C) pH ∼7.0, and (D) pH ∼9.0.

Next, it is imperative to unfold the kinetic and mechanistic properties by taking advantage of the effect of concentration as well as scan rates at different pH regarding arsenic oxidation. Here, employing the modified Langmuir eq. (5) [44], the adsorption equilibrium constant was determined by plotting [As(III)]/j_p_ against [As(III)] as shown in Fig. 5(A-D).

(5) $$\frac{\left[As\left(III\right)\right]}{j_{P}}=\frac{1}{bj_{p,\max}}+\frac{\left[As\left(III\right)\right]}{j_{p,\max}}$$

Where, *b* implies the adsorption equilibrium constant, *j*_*p*_ and *j*_p. max_ represent the current density and maximum current density, respectively, at peak potential. Using the slope and the intercept of the plots in Fig. 5(A-D), the adsorption equilibrium constant was determined as tabulated in Table 2. The evaluated adsorption equilibrium constant in acidic medium was found to be almost two times more than in basic medium, which implies the strong affinity of the arsenic species in acidic medium.

**Table 2:** Typical kinetic properties for As(III) oxidation at different pH using Au electrode.

Kinetic properties at different pH			
pH	*b* (molL^−1^) × 10^5^	ΔG^0^ (kJmol^−1^)	$\Gamma_{c}$ (molcm^−2^) × 10^−9^
3.0	3.21 (±0.8)	−31.15 (±0.5)	1.58 (±1.3)
5.0	2.78 (±0.6)	−30.56 (±0.4)	1.43 (±1.2)
7.0	1.91 (±0.6)	−29.89 (±0.6)	1.53 (±0.8)
9.0	1.61 (±0.5)	−28.73 (±0.5)	0.92 (±0.7)

Later, employing the equilibrium constant for each certain pH values, the change of the Gibbs free energy due to the adsorption of As(III) ions on Au surface, ΔG^0^ (=−*RT* ln *b*) was also estimated (see Table 2). The observation implies that in basic medium, the Gibbs free energy change is higher than that of acidic medium pertaining to the oxidation of As(III) and the reactions require more driving force for the respective oxidation reaction as a matter of fact that the negative hydroxyl ions may repeal the negative As_3_O_4_^−^ ions during the continuation of the reaction. Afterwards, the amount of As(III) adsorbed on the gold electrode surface was determined for the each pH values from eq. (6) [45],

(6) $$\dot{j_{p}}=\frac{n^{2}F^{2}\Gamma_{c}\nu }{4RT}$$

**Fig. 6:**
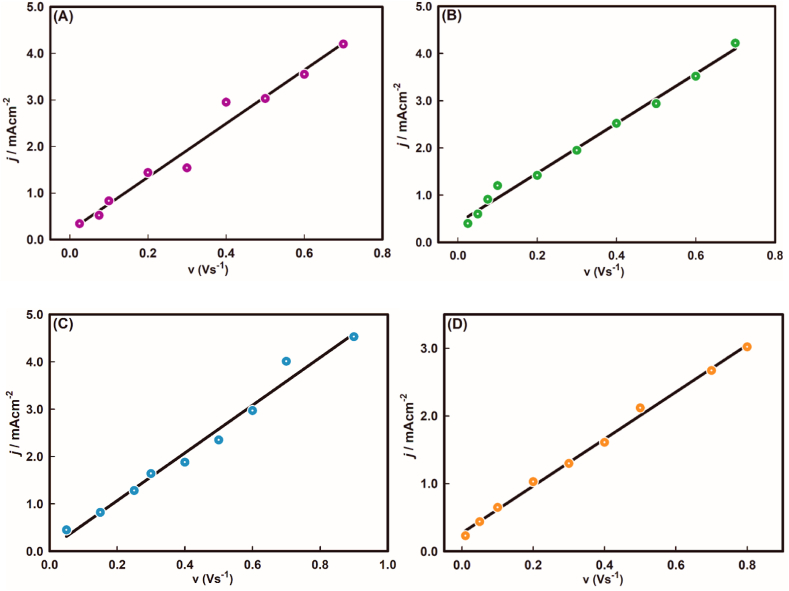
Dependency of current density, *j* against scan rate, *v* at (A) pH ∼3.0, (B) pH ∼5.0, (C) pH ∼7.0, and (D) pH ∼9.0.

Where, $\dot{j_{p}}$ indicates the peak current density, *n* indicates the number of electrons transferred, $\Gamma_{c}$ indicates the surface coverage of the electrode reaction (molcm^−2^), *F* indicates the Faraday constant (96485 C mol^−1^), *R* indicates the gas constant (8.314 J K^−1^ mol^−1^), *v* indicates the scan rate (Vs^−1^) and *T* indicates the temperature (298 K). Plots of peak current density against the scan rate were drawn (see Fig. 6(A-D)) to calculate the surface concentration of the electrode from the slope values of each plot. The calculated values of $\Gamma_{c}$ are tabulated in Table 2. It is revealed that with the rise in pH values, the surface coverage decreases.

However, for the irreversible surface electrode process of As(III) oxidation on Au surface, eq. (7) [46] could be used for the determination of transfer co-efficient from the slope of *E*_*p*_ against natural logarithm of scan rate,

(7) $$E_{p}=E^{0}+\frac{RT}{\beta nF}\ln\left(\frac{RTk^{0}}{\beta nF}\right)+\frac{RT}{\beta nF}\ln\left(\upsilon \right)$$

Here, *E*^*0*^ is the formal potential, *β* is the transfer coefficient for an anodic process, *n* is the number of electrons transferred in the rate determining step, which is about to 2 and *k*^*0*^ is the standard heterogeneous rate constant.

**Fig. 7:**
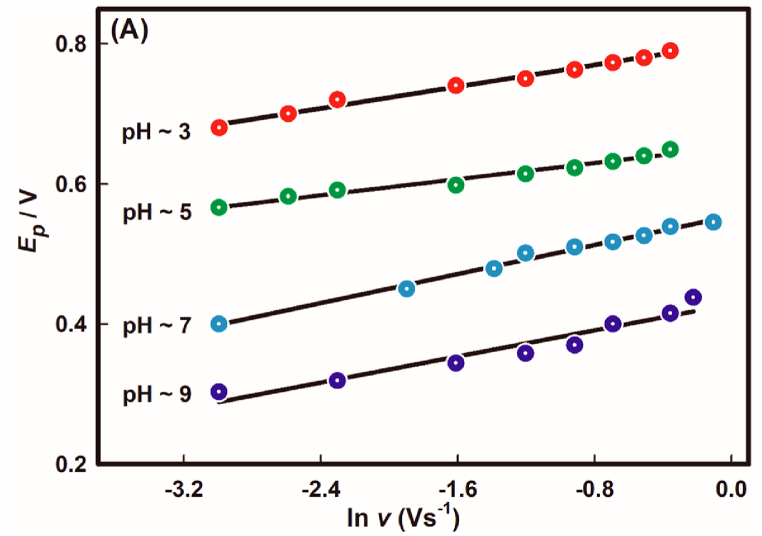
A plot of peak potential against the logarithm of scan rate of variable pH at 100 mVs^−1^ scan rate. The experimental conditions are same as mentioned in Fig. 3.

The slopes found from Fig. 7 were employed to determine the anodic transfer co-efficient, β, of the each pH values pertaining to the oxidation of As(III) using eq. (7). The β values were evaluated to be 0.85, 0.78, 0.45 and 0.41 for pH ∼3.0, ∼5.0, ∼7.0 and ∼9.0, respectively. The values indicate that the oxidation of As(III) on Au surface follows step-wise mechanistic pathway for acidic conditions (pH ∼3.0 and ∼5.0) [47]. Conversely, for neutral (pH ∼7.0) and basic medium (pH ∼9.0), the reaction pathway followed concerted mechanism for the concerned oxidation process [48].

**Fig. 8:**
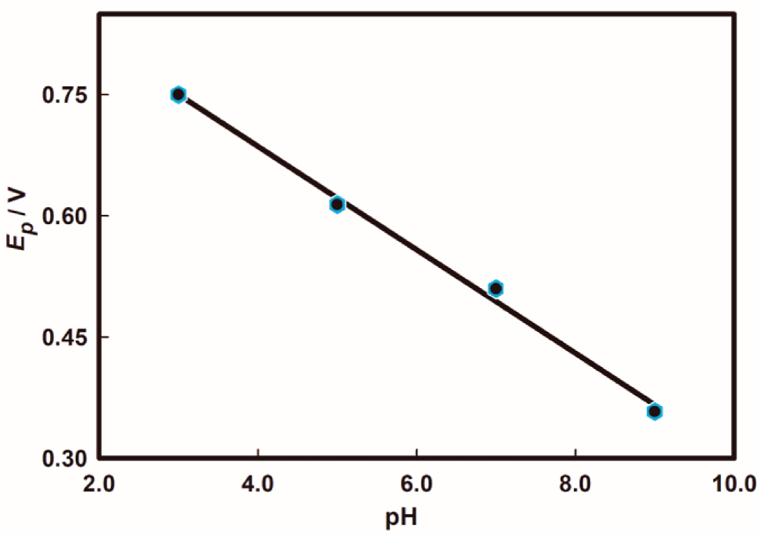
A plot of peak potential (*E*_*p*_) against pH for As(III) oxidation on Au electrode surface.

Later, the effect of pH on the potential variation for the oxidation of As(III) was also investigated to look into the proton involvement pertaining to the electrode reaction. It is noticeable from Fig. 8 that the peak potential of As(III) oxidation decreases with the increment of pH.

A plot of peak potential, *E*_*p*_, against pH renders a good linear relationship with eq. (8),

(8) $$E_{p}\;(V)=-0.063\;pH+0.942;\;\left(r^{2}=0.99\right)$$

Here, the slope found from the relationship is nearly the theoretical value of 59.0 mV pH^−1^. According to the equation [49], −59.0 m/n = −63.0, where m is the proton participating in the electrode reaction of the As(III) oxidation and n is the number of electron transferred in the oxidation. Note that the ratio of the equation indicates that the number of protons (m) accompanied same number of electrons transferred during the electrode process which was determined to be m = n = 2. That means two electrons were associated with two protons during the electrode reaction of the As(III) oxidation.

## 4 Conclusion

A systematic kinetic exploration on As(III) oxidation studies were performed using Au electrode from pH ∼3.0 to ∼9.0. The oxidation of As(III) on Au surface is a purely adsorption-controlled process. On top of that, the adsorption equilibrium constant for acidic medium was found almost two times more of basic medium pertaining to the oxidation. The number of electron transfer was also verified that two protons accompanied two electrons in the respective oxidation. Moreover, the oxidation reaction adopts a stepwise pathway for acidic medium, meanwhile for neutral and basic medium, the reaction adopts a concerted pathway. Above all, an acidic medium was found to be more suitable for the concerned oxidation. On extension, further kinetic studies can be performed in gold modified electrode surface for arsenic oxidation at different pH environment by varying temperature.

## Author contribution statement

Mohebul Ahsan: Conceived and designed the experiments; Performed the experiments; Analyzed and interpreted the data; Wrote the paper.

Muhammad Zobayer Bin Mukhlish: Contributed reagents, materials, analysis tools or data.

Nazia Khatun: Analyzed and interpreted the data.

Mohammad Abul Hasnat: Conceived and designed the experiments; Contributed reagents, materials, analysis tools or data.

## Funding statement

Mohammad Abul Hasnat was supported by Shahjalal University of Science and Technology [PS/2022/1/01], Ministry of Science and Technology, Government of the People’s Republic of Bangladesh [SRG-223537, Year 2022], Ministry of Education, Government of the People's Republic of Bangladesh [PS 20201512].

## Data availability statement

Data will be made available on request.

## Declaration of interest’s statement

The authors declare no conflict of interest.
