# Supplementary material for: pH dependent electro-oxidation of arsenite on gold surface: Relative kinetics and sensitivity
Source: Heliyon. 2023 Mar 6;9(3):e14192. doi: 10.1016/j.heliyon.2023.e14192 (PMC10023909; doi:10.1016/j.heliyon.2023.e14192)
Supplement: Multimedia component 1 [file mmc1.docx]

**pH dependent electro-oxidation of arsenite on gold surface: Relative kinetics and sensitivity**

**Mohebul Ahsan^a^, Muhammad Zobayer Bin Mukhlish^b^, Nazia Khatun^c^, Mohammad A. Hasnat^a*^**

*^a^Electrochemistry & Catalysis Research Laboratory (ECRL), Department of Chemistry, School of Physical Sciences, Shahjalal University of Science and Technology, Sylhet-3114, Bangladesh*

*^b^Department of Chemical Engineering & Polymer Science, Shahjalal University of Science & Technology, Sylhet-3114, Bangladesh*

*^c^Industrial Physics Division, Bangladesh Council of Scientific and Industrial Research (BCSIR)*

*^*a^Corresponding author*: E-mail: [mah–che@sust.edu](mailto:mah–che@sust.edu), [mahtazim@yahoo.com](mailto:mahtazim@yahoo.com)


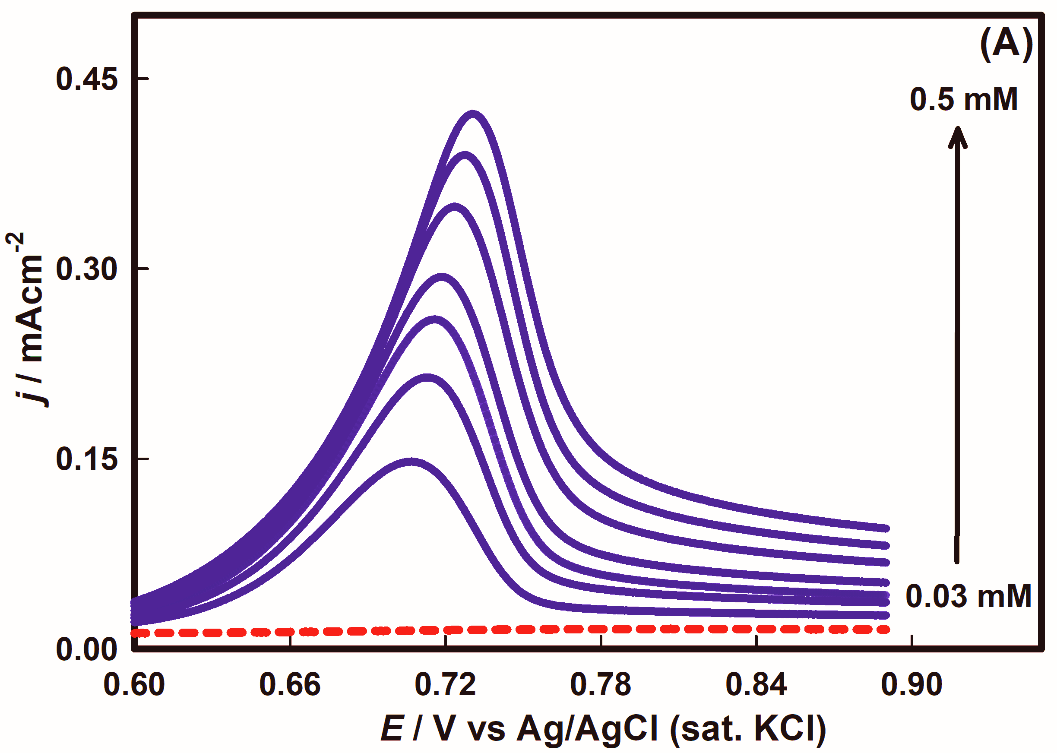

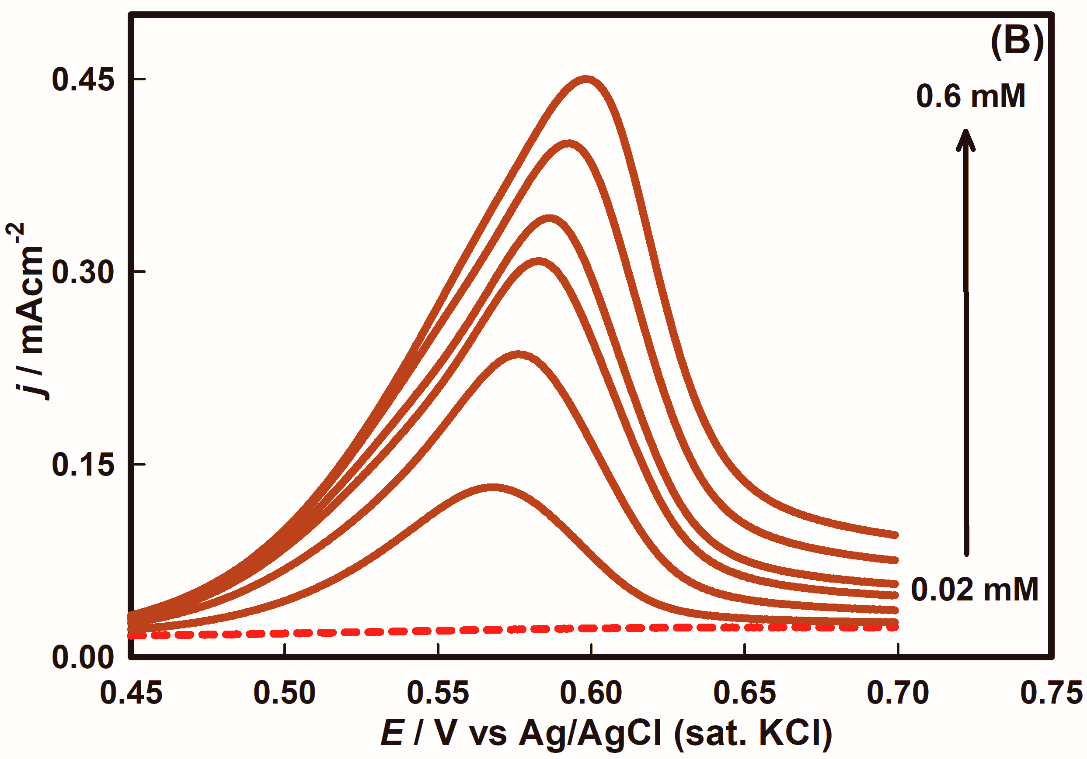


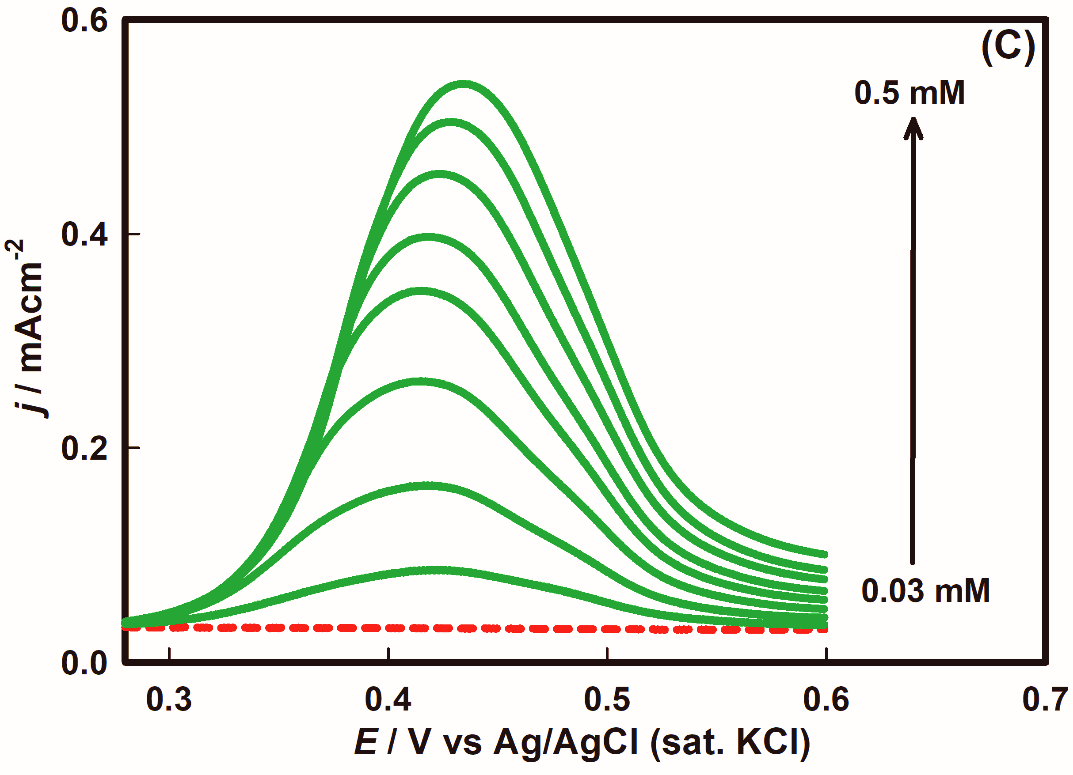

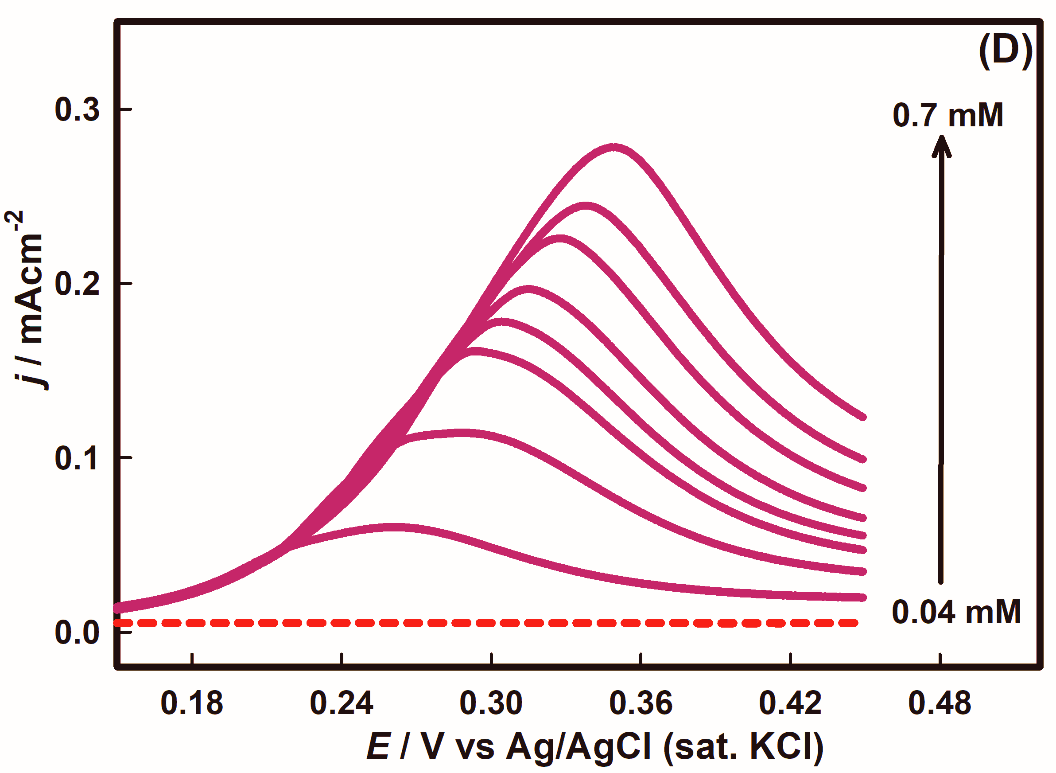


**Fig. S1. Concentration dependent linear sweep voltammograms at (A) pH ~3.0, (B) pH ~5.0, (C) pH ~7.0, and (D) pH ~9.0 at 100 mVs^-1^ scan rate.**


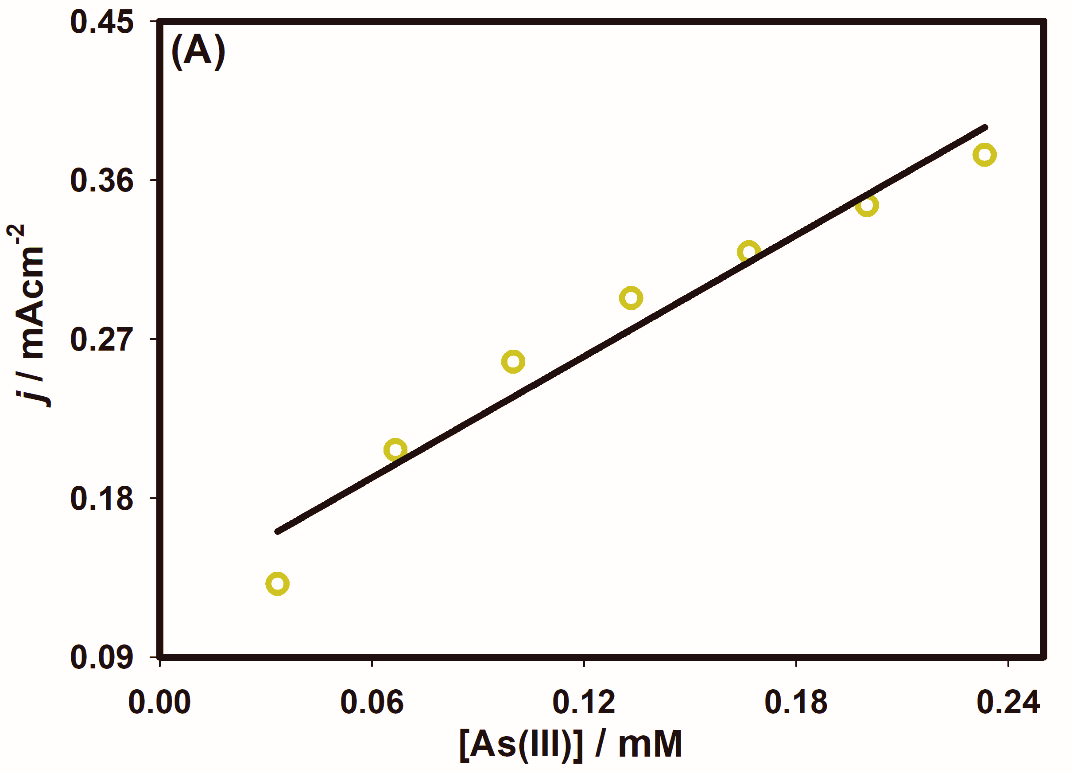

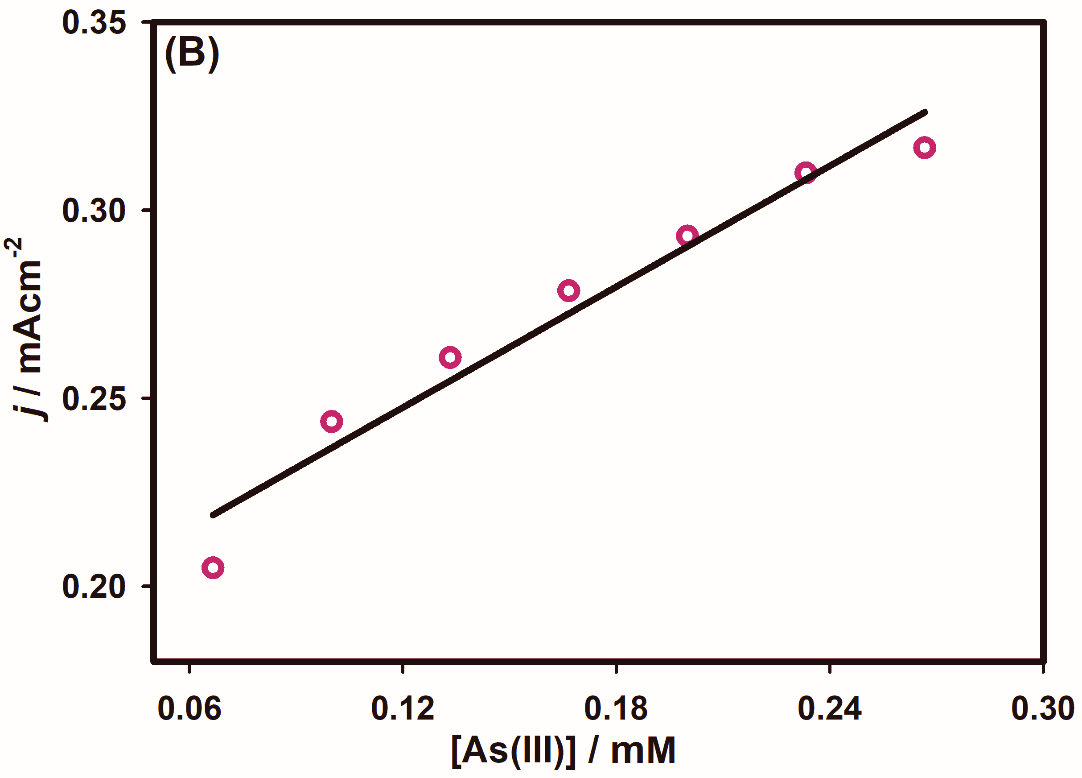


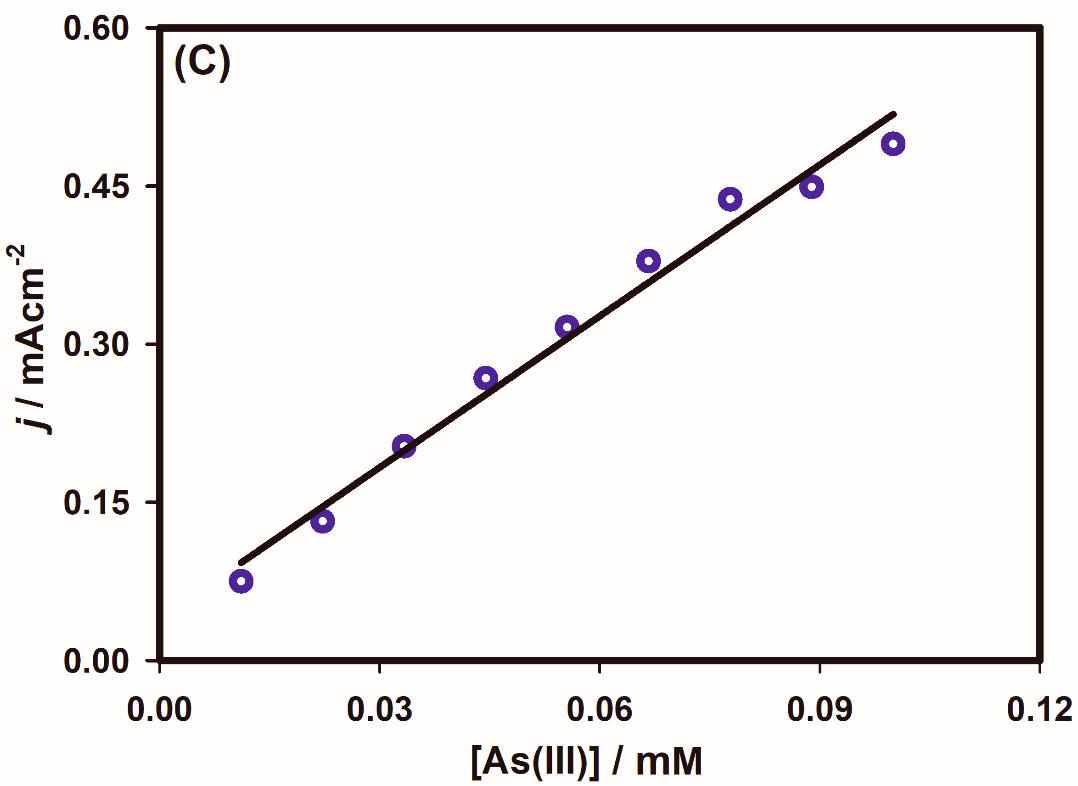

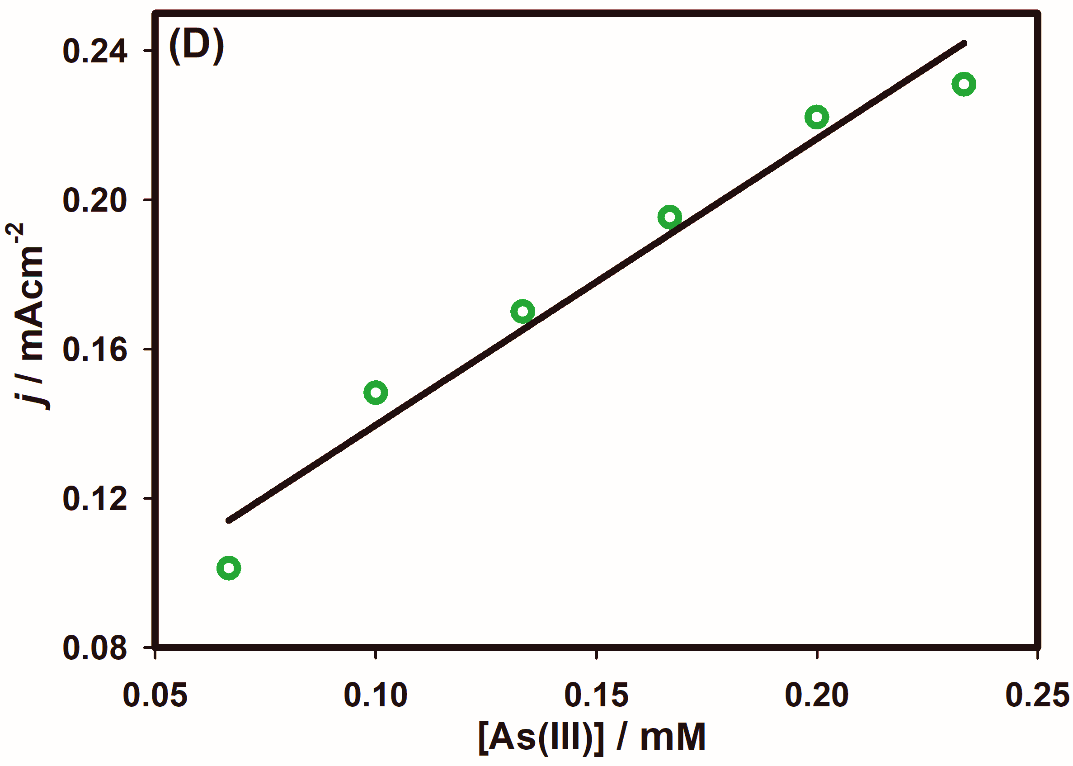


**Fig. S2. Plots of current density, *j* vs As(III) concentration at (A) pH ~3.0, (B) pH ~5.0, (C) pH ~7.0, and (D) pH ~9.0.**
